# Frequency of *Human Papillomavirus* Genotypes among Women with Genital Wart Using Molecular Hybridization Methods 

**DOI:** 10.30699/ijp.2024.2013097.3182

**Published:** 2024-10-29

**Authors:** Zahra Shahriyari, Mohammad Niakan, Zahra Soleimani

**Affiliations:** 1 *Department of Microbiology, Faculty of Medicine, Shahed University, Tehran, Iran*; 2 *Department of Obstetrics and Gynecology, Nephrology and Urology Research Center, Baqiyatallah University of Medical Sciences, Tehran, Iran*

**Keywords:** Human papillomavirus, Genotype distribution, Genital warts, Molecular hybridization

## Abstract

**Background & Objective:**

*Human papillomavirus* (HPV) is one of the most common sexually transmitted infections worldwide, which can lead to virus-related cancers. This study aimed to investigate the frequency of HPV genotypes in women with genital warts referred to available laboratories in Tehran by molecular hybridization method.

**Methods:**

This cross-sectional descriptive study was conducted on the genital warts of 67 women aged 20-50, who were referred to the clinics of Afshar, Namad, Mani, and Al-Mohammed in Tehran province. Viral DNA was extracted using Add prep genomic DNA extraction kit, and genotyping was studied using HPV Direct Flow CHIP Kit. Data were analyzed by GraphPad Prism software.

**Results:**

HPV was reported to be positive in all cases. The most common low-risk genotype involved was type 6, with 30 cases (44.77%), and the most common high-risk genotype involved was type 16, with 4 cases (5.97%) in the total population. Among the patients examined, there were 16 cases with multiple infections.

**Conclusion:**

The results of this study showed that low-risk genotypes may be responsible for majority of the genital warts. High-risk genotypes and simultaneous infection with several genotypes could also be common in genital wart samples. Therefore, controlling HPV infection is important, especially in patients with high-risk genotypes. HPV genotyping should be considered in diagnosis and prevention of HPV-related cancers.

## Introduction

*Human papillomavirus* (HPV) is associated with the development of several benign and malignant neoplasms (1). HPV is responsible for 4.5% of the cancer cases worldwide (2). There are 80 million known HPV-positive individuals in the United States (2). These nonenveloped particles are double-stranded circular DNA viruses that affect about 50–70% of sexually active individuals and account for more than 5% of all cancers and approximately 50% of the malignancies worldwide. It has been proven that approximately 225 types of HPV have been identified, which can be divided into five groups, including α, β, γ, µ, and ν, and approximately 15 are high-risk HPV-α viruses (3).

Low-risk genotypes usually give rise to precancerous lesions such as warts, and include different types such as 6, 11, 40, 42, 43, 44, 54, 55, 61, 62, 67, 69, 70, 71, 72, 81, 84 and 89 (4-6). Furthermore, high-risk genotypes such as 16, 18, 26, 31, 33,35, 39, 45, 51, 52, 53, 56, 58, 59, 66, 68, 73, and 82 tend to cause cancers such as cervical cancer (100%), anal cancer (88%), vaginal cancer (78%), pharynx and oral cancer (30-70%), male genital malignancy (51%) and vulva cancer (less than 25%) and 70% mentioned cancers are due to common high-risk types such as 16 and 18 (7-10).

Studies have indicated that HPV genotypes are responsible for 97% of all cervical cancer cases, and HPV 16 and 18 are in charge of 90% of HPV-related cancers (11).

These types of viral infections are often transferred through direct contact, skin scratches, sexual contact, and birth canal. Hence, vaccines could help in protection of HPV strains that are more likely to cause genital warts or cervical cancer (12). Also, in most cases, the immune system defeats HPV infection before it causes warts, and HPV infection is often self-limiting, but in 10-15% of cases, it could lead to cervical intraepithelial neoplasia (CIN-2) or even advanced stages (13).

Prevalence of cervical cancer has decreased by as much as 65% between 1970 and 2011 in various Western nations, including Norway (14). In addition, rates have also decreased in high-risk regions like China as well (15). Contrary to the positive trends mentioned, in various countries, including Finland, the UK, Denmark, and China, prevalence of cervical cancer has been rising among younger generations (16). The HPV vaccine holds the potential to alter the epidemiology of the disease, but current HPV vaccination rates are still too low to prevent disease spread (2). 

Genital warts are one of the most common sexually transmitted diseases, which are mostly transmitted through sexual contact and vertical transmission. Approximately 1% of the sexually active population present with symptomatic genital warts (17).

In general, 10.4% of women worldwide with normal cervical cytologic results also present with HPV infection. Less developed areas have shown higher prevalences as follow: 22.1% in Africa and 20.4% in Central America and Mexico, compared to 11.3% in North America, 8.1% in Europe, and 8.0% in Asia. According to a study performed in 2010 on women with normal cytology, women under 25 demonstrated the highest prevalence of HPV (23.2%) (18).

In 2014, 3.71 million Iranian women were infected with HPV, which indicates a rapidly increase over the years. Currently, 25 to 30 million women older than 15 years of age are at risk for cervical cancer in Iran. However, HPV vaccines are currently not applied in the country's national vaccination program, although individuals can purchase them at their own expense. It must be emphasized that vaccinations do not exclude the need for effective screening. Therefore, both vaccinated and nonvaccinated women need to undergo HPV screening (19).

Information about HPV genotyping distribution allows for a better understanding of the correct distribution of the burden of HPV-related diseases and their impact. In various studies conducted on routine pap-smear samples in Iran, HPV-16 and -18 represented as the main detected genotypes (20-22), However, no fixed pattern of the HPV genotypes was observed even in different parts of the same province. 

The other study, Using the HPV Direct Flow CHIP System, assessed the prevalence of low risk- and high risk-HPV in 12,076 Iranian women who have undergone standard cervical cancer (CC) screening and HPV DNA typing. The following ten high-risk HPV genotypes were prevalent: HPV 16 (552, 16.98%), HPV 18 (250, 7.69%), HPV 31 (242, 7.45%), HPV 45 (183, 5.63 %), HPV 52 (286, 8.8%), HPV 39 (248, 7.63%), HPV 51 (213, 6.55%), HPV 66 (171, 5.26%), HPV 68 (180, 5.54%), and HPV 58 (157, 4.83%). Among all subjects, HPV 16 was the most prevalent high-risk genotype, but HPV 26 was less common. Furthermore, among Low-risk-HPVs, the top three most common genotypes were HPV 6, 11, and 62/81 (4).

The research yielded an HPV infection rate of 38.68%, of which 17.41%, 32.03%, and 50.56% were considered to be high-risk, low-risk, and multiple high- and low-risk infection rates. The most prevalent infections among the 36 identified High-risk and low-risk-HPV subtypes were HPV 6, 16, 11, 62/81, 52, and 54, in that order. These accounted for almost 47% of all HPV types (4). 

Unfortunately, a precise and comprehensive information on HPV prevalence is not available for the Iranian women population, and due to increasing progress in screening techniques and vaccination programs in advanced societies, the differences in HPV prevalence between women in developed and developing countries are significant . In addition, lack of knowledge and information about HPV virulence and HPV vaccine and its complications are reasons for not using the vaccine. According to the fact of investigated in this field, although part of this infection is due to socioeconomic limitations, the epidemiological part of HPV genital warts prevalence remains unknown in recent years. Therefore, punctual diagnosis and treatments are important. According to the results, it is necessary to obtain epidemiological information and molecular characteristics provided to health-treatment departments (23). Consequently, by obvious effort regarding know the variety of new genotypes, we aimed to determine the prevalence of low and high-risk HPV genotypes among women with genital warts in 6 months, which was carried out in Afshar, Namad, Mani laboratories and Al-Mohammed Clinic in Tehran in March 2023 and investigated with new method of HS12 *in situ* hybridization.

## Materials and Methods

This descriptive-sectional study was conducted on 67 women with genital warts aged 20-50 i , who were referred to Afshar, Namad, Mani, and Al-Mohammed clinics in Tehran in March 2023, to detect presence of papillomavirus and determine genotypes. A gynecologist stamped genital warts placed them in special liquid-based cytology (LBC) solutions and sent them to the mentioned laboratories. A total of 67 clinical samples were examined. Furthermore, consent was obtained from all the patients, and none of them declined to participate in this study. A questionnaire containing patient demographic information, sexually transmitted diseases background, smoking, and alcohol was given to the patients. As soon as the samples arrived at the laboratory, they were stored in -20°C.

After preparing the samples, the clinical samples were mixed with 200 μL of lysis buffer and 20 μL of proteinase K, and then incubated at 56 ◦C, and samples were mixed with 200 μL of Binding buffer and then incubated at 56˚C for 10 minutes. The solution was mixed with 200 μL of ethanol and transferred supernatant into the column, then centrifuged at 13000 rpm for 1 minute. The column was placed in the new collection tube, 500 μL of washing buffer one was added to the column and centrifuged at 13000 rpm for 1 minute. Then, 500 μL of washing buffer two was added to the column and centrifuged at 13000 rpm for 1 minute. The centrifuge was centrifuged at 13000 rpm for 1 minute to dry the filter membrane. The column was put into a clean and sterile centrifuge tube, and 50 μL of elution buffer was added to the column.

DNA was extracted by using the “Add prep genomic DNA” extraction kit (AddBio company) based on its instructions. Genome reproduction steps were carried out in a thermocycler machine, and an “HPV direct flow chip kit” (Master Diagnostic, France) was used to determine the virus genotypes. It could be mentioned that this kit was able to identify 35 HPV genotypes at the same time.

DNA was duplicated by “PCR based on a hybridization amplification kit” from the agreed region of the viral L1 protein, and reverse dot blot Hybridization was used to detect the genotypes of the virus. In this method, there are two dotting blots for each genotype and control object, which are complementary and confirm each other. Therefore, the results are reliable and minimize false positive and negative results. Ultimately, by observing and interpreting the microchips, the results were recorded and analyzed by using demographic information, and GraphPad Prism.

## Results

In this study, HPV was reported to be positive in all 67 cases. Also, it was shown that low-risk genotypes to be responsible for majority of the genital warts (53.73%). High-risk genotypes and simultaneous infection with several genotypes were also common in genital wart samples (46.26%).

**Fig. 1:**
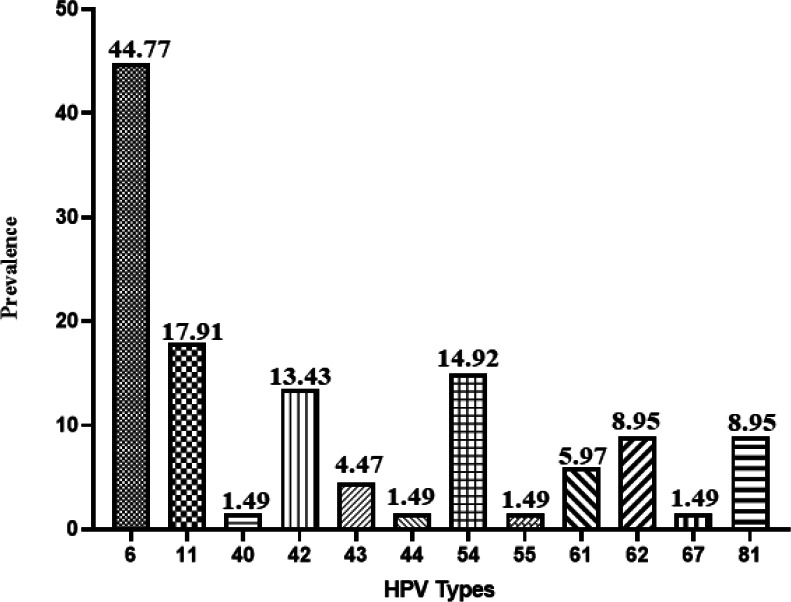
Abundance of the low-risk genotypes

Distribution of the low-risk-HPV genotypes in this study basically resembles the other parts of the world, and the most common low-risk type of HPV was 6 (30 cases,44.77%) in the total population (Figure 1). Other genotypes abundances were: 11, 54, 42, 43, 61, 81, 62, 67, 44, 40, and 55.

**Fig. 2:**
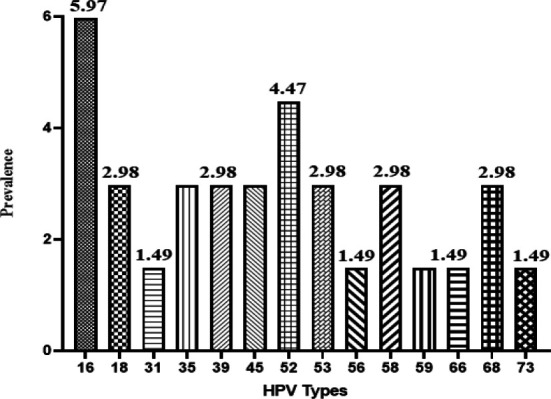
Abundance of the high-risk genotypes

Also, the most common high-risk genotype involved was type 16 (4 cases, 5.97%) in the entire population, and HPV52 was far more common than HPV18 and made the second-highest percent (4.47%) in samples (Figure 2). Other genotypes to genotypes abundances were 52, 68, 58, 53,45, 39, 35, 18, 31, 56, 59, 66, and 73.

**Fig. 3:**
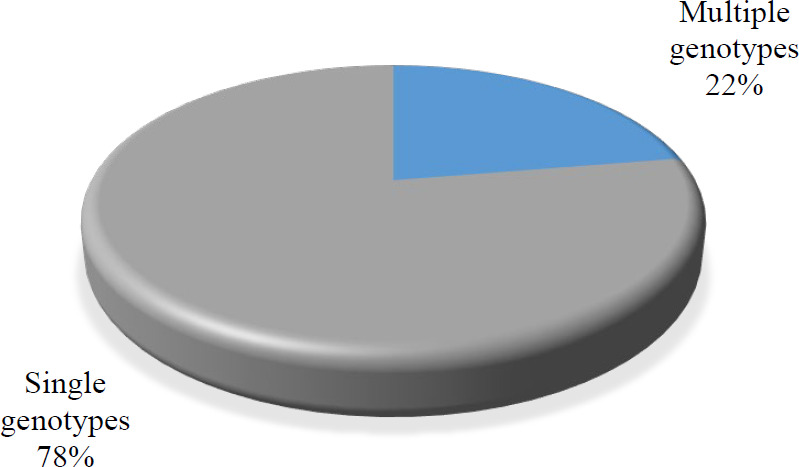
Abundances of patients with single and multiple genotypes

Among the examined patients, 16 samples with multiple infections were observed. These multiple genotypes include co-infection with genotypes such as (52-54), (18-61- 42), (81-11-62-68-53), (52-6-67-54-42-40), (73-6), (52-54), (59-6), (39-53-6), (58-55-44), (18-39-11), (35-45-81-62-42), (56-45-58-54-43-42), (16-42), (58-81-62), (68-53-81-62), (16-6), and (16-6). In summary, 22% of the patients were infected with multiple genotypes, and a high proportion of the women harbored single HPV infections (78%) (Fig 3). 

**Fig. 4:**
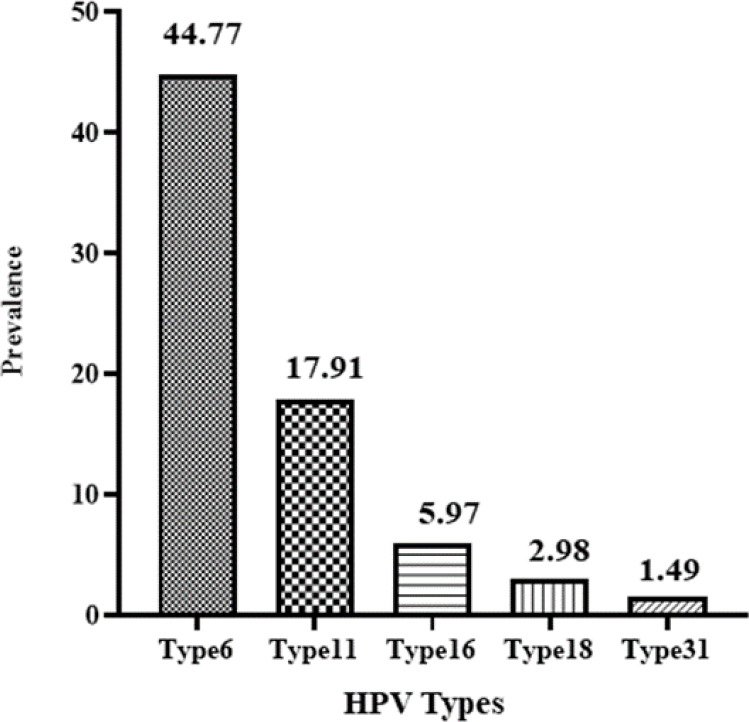
Percentage of common HPV genotypes in the population of this study

**Fig. 5:**
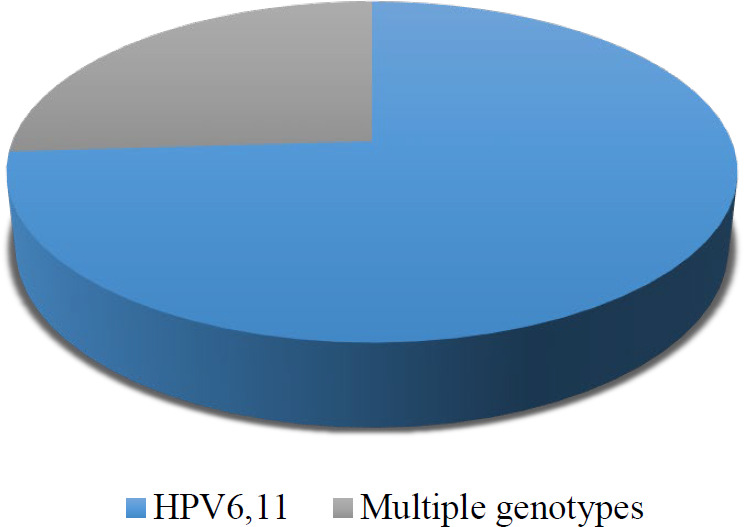
Population segregation by common HPV 6 and 11 genotypes and multiple genotypes

Based on recent studies, 5 genotypes, including 6, 11, 16, 18, and 31, have been identified as common genotypes among the patients with genital warts (24). In the present study, five common HPV genotypes were identified such as type 6 (44.77%), type 11 (17.91%), type 16 (5.97%), type 18 (2.98%), and type 31 (1.49%) (Fig 4). HPV 6 and 11 together accounted for 62.68% of HPV infections (Fig 4). Also, multiple HPV genotypes were recognized in 22% of the samples. Abundance of high-risk HPV genotypes, including 16 and 18, was low and 8.95% in the total population. Consequently, the predominant HPV genotypes found in this study among women with genital warts in Tehran were low-risk HPV, including 6 and 11 (Figure 5).

**Fig. 6:**
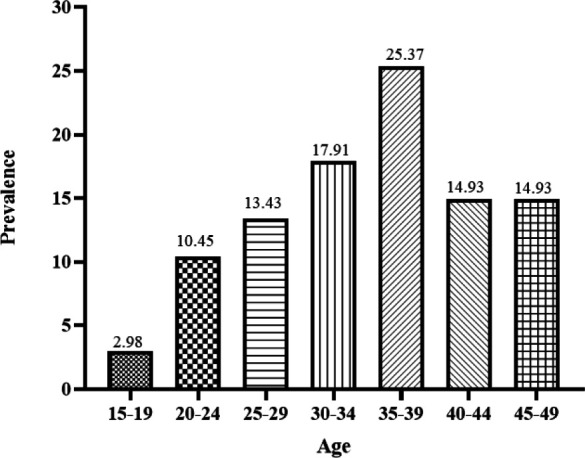
Distribution of the studied population in terms of age

The highest prevalence of HPV in the present study was between the ages of 35-39, and it is up to 25.37% (Figure 6). With increasing age, the HPV prevalence decreased. In women over 30 years, the prevalence of HPV is about 15%.

**Fig. 7:**
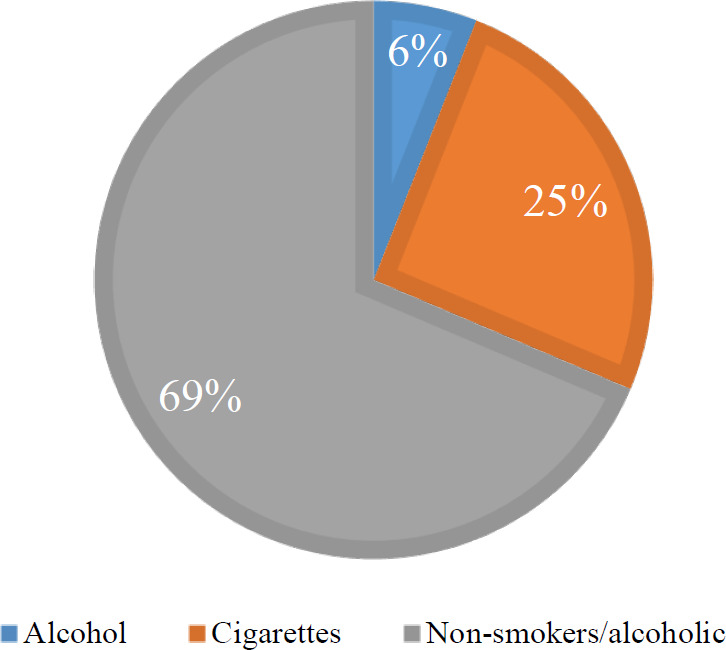
Separation of the population in terms of smoking or alcohol use

In general, the population could be divided into two general categories: smokers or alcohol users and non-users. About 25% of the investigated patients are smokers, and 6% are alcohol consumers (Figure 7). These patients had more high-risk genotypes, although these differences were not statistically significant between smoking and alcohol use and HPV infection.

**Fig. 8:**
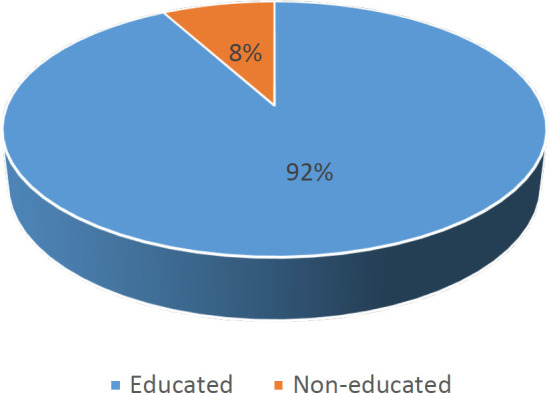
Separation of the population based on the education level.

Moreover, the population is divided into two categories: educated and non-educated. About 92% of the studied cases were educated (Figure 8). Studies illustrated that the level of education is related to HPV prevalence (25).

For instance, a study in the United States displayed that women with higher education were less likely to suffer from HPV infection. This link could be because people with higher education have more information about methods of prevention of sexually transmitted diseases and the risks of smoking and alcohol consumption and pay more attention to their physical health. Also, people with higher education might work in occupations that require attention to physical health and, consequently, make more efforts to prevent sexually transmitted diseases and use contraceptive methods. Nevertheless, these studies are contrary to our studies.

**Fig. 9:**
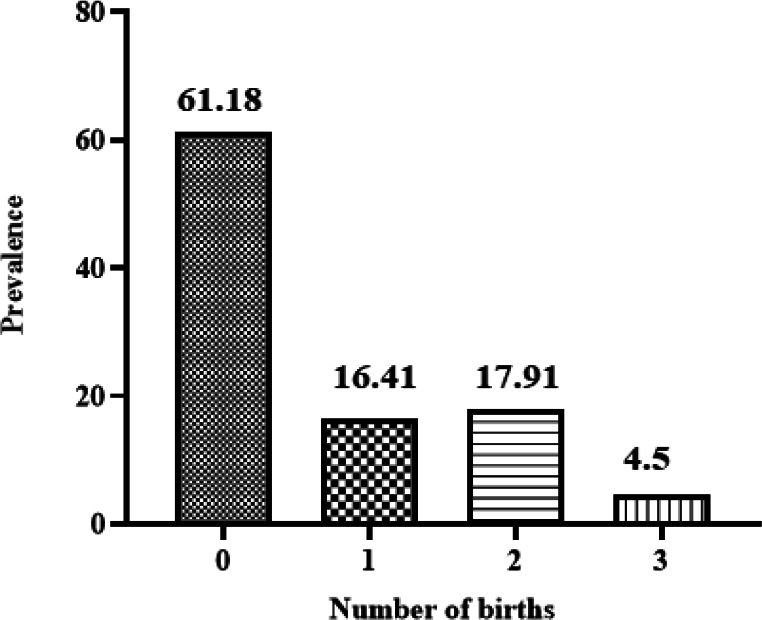
Separation of the population based on birth frequency.

This study showed no direct link between birth frequency and HPV infection (Figure 9). However, some studies have proved that women who have given birth more than 3 times might be at higher risk for HPV infection (26). Thus, these subjects need further research.

**Fig. 10:**
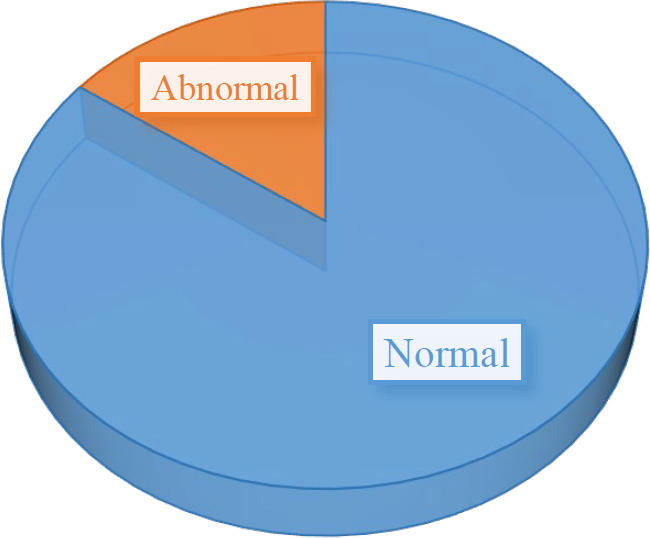
Correlation between the latest pap smear test result and HPV

Also, in this investigation, the last pap smear test of 85% of the subjects was normal (Figure 10). Therefore, a pap smear test is not able to be used as a screening method for HPV infection in women.

## Discussion

As mentioned before, HPV is the most common cause of sexually transmitted infections worldwide, with a lifetime risk of infection in men and women of about 50%. Persistent infection with high-risk HPV is associated with several human cancers, especially cervical cancer (27). 

Chronic high-risk HPV infection can produce cervical cancer and increase the risk of cancer in the cells (28). 22.1% of Africans, 11.3% to 20.4% of Americans, 8.1% of Europeans, and 8.0% of Asians were found to be HPV positive (29). Benign lesions give patients a great deal of anxiety and agony, even though low-risk-HPV subtypes do not produce serious, life-threatening cancers (4). 

In the past several years, a great deal of research has been done on the prevalence of HPV in women. However, as our study of the literature indicates, not much research has investigated Iranian women's High-risk and Low-risk-HPV genotypes (30, 31).

As a result, the current study was conducted to determine the frequency of human papillomavirus genotypes in females. Because genital warts are a common sign of HPV infections and have been proven to be highly prevalent in prior research, we focused on the individuals who had them. 

In this study, HPV was reported as positive for all 67 cases with genital warts. In another study in Iran, the HPV prevalence among women aged 15 to 59 was 2.8% (108 out of 2526). The high rate of this statistic in our study compared to other studies could be because patients examined in our study had genital warts, which is a characteristic of HPV infection. In contrast, in other investigations, sampling was done in a clustered manner (32).

Our results showed 46.26% and 53.73% of patients had high and low-risk genotypes, respectively. Similar to our study, Chalabiani *et al.*, the prevalence of high-risk HPV in different regions of Iran was reported at 67.2% (33).

In this study, most of the women who had both low and high-risk genotypes were also less than 30 years old. 

The results are in line with earlier research showing that HPV was more common in younger people, specifically those between the ages of 20 and 29(34). 

In many countries, the highest prevalence of this virus is usually seen in younger age groups and factors such as the young age of starting sexual activity, the young age of marriage, unusual sexual culture, economic status, and smoking are said to be the reason for this issue. For instance, in results that were reported by Coser *et al.*, a high prevalence of HPV infection and its persistence in women under 30 years of age were observed in a region of Brazil that also suffers from financial poverty (35). 

Some women typically eradicate the infection relatively rapidly because of their strong immune systems, as well as their stability in both their sexual and financial lives; this helps to explain why the prevalence of HPV declines beyond the age of 25. Therefore, eliminating cervical precancerous lesions found during screening could guard against contracting fresh HPV infections (36). Together, these results, which are consistent with earlier research, indicate that the level of contamination has gradually decreased with increasing age (37). The natural history predicts that the prevalence of HPV will peak in younger women and then fall as they get older. However, according to Kim et al.'s study, the second peak in HPV prevalence occurred between 40 and 49. This finding is interesting because it suggests that both aging-related immune system decline, and improper screening of the lesions could cause the recurrence. A new generation of broad-spectrum vaccinations or, to a lesser extent, policymaking for bolstering immunization campaigns in these regions will be made easier with knowledge of the genotypic prevalence in that region (38).

Our findings suggest that a total of 26 HPV genotypes, including 14 high-risk types (18, 53, 52, 68, 73, 59, 35, 39, 58, 45, 56, 16, 66, and 31), as well as 12 low-risk types (6, 11, 42, 61, 62, 81, 43, 54, 40, 67, 44 and, 55) were identified. Among high and low-risk genotypes, HPV 6 was higher than other types, which indicates its abundance. The same result has been reported in different regions of Iran and worldwide (39, 40).

The genotypes abundance of 6, 11, 16, and 18 among women examined in this study was 44.77%, 5.97%, 2.98%, and 2.98% respectively. In a similar study that was conducted in Khorasan City, the prevalence of type 6 (50%), type 11 (10%), and type 16 (15%) was like our study, and the most common type was type 6 (41). In addition, based on recent studies, HPV 16 was the most common type of high-risk HPV genotype in all reviewed cases; it has the highest potential for carcinogenesis and is a key candidate for HPV vaccines (42). Other researchers in Iran also reported that HPV 18 was the second most common genotype after HPV16 (43). 

Generally speaking, data indicates that HPV18 is more prevalent than HPV16 globally, while incidence varies by location (7, 11, 12, 44, 45, 46, 75).

Contrary to the results, the present study illustrated that HPV 52 is the second most high-risk genotype. Also, results of other findings described genotypes 31, 35, 39, 53, and 58 as well as 16 and 18 (10, 42, 46). 

Furthermore, our results were in line with the evidence presented by Sudenga *et al.* (47), which showed that HPV 6 and 11 are frequently detected in genital warts in patients.

The most common High-risk HPV types in this study were HPV 16, 52, 18, 39, 31, 51, while low-risk-HPV types were HPV 6, 11, 62/81, 54, 42, 40, and 44/55. According to Mobini *et al.*, consistently, 16.6% of the 10,226 subjects who underwent assessment had HPV-16, and 9.6% had HPV-52. This study found that HPV 16 and 52 are the most prevalent high-risk HPV varieties in the Iranian population. 

Furthermore, human papillomavirus genotype 52, or HPV52, is the sixth or seventh most prevalent HPV genotype globally linked to cervical malignancies (48, 49). However, studies conducted in East Asia have shown that HPV52 is ranked substantially higher. For example, in Hong Kong, HPV52 was the second most common HPV genotype found in cervical intraepithelial neoplasia 2 (CIN2) and CIN3 and the third most common HPV genotype found in cases of squamous cell carcinoma (50). Moreover, HPV52 was the most prevalent genotype in instances of cervical cancer in Shanghai (51) and the second most prevalent in cases in Taiwan (52) and Japan (53), which is consistent with our research. 

These differences in HPV genotype distribution indicate the divergence of HPV prevalence in different regions (10). 

The fundamental cause of this spatial concentration of disease attribution is yet unknown. High-risk genotypes are linked to a malignant transformation of cells, such as oropharyngeal and anogenital malignancies, whereas low-risk genotypes are typically associated with genital warts and respiratory tract papilloma (54).

Consequently, variations in world locations, study populations, sexual practices, health status, and HPV testing techniques can lead to disparities in prevalence between studies, which can significantly impact the findings.

Also, outcomes of research showed that, among patients with both low and high-risk genotypes, the abundances of HPV 16 and 52, as well as 6 and 11, were higher than those of other genotypes. In this research, multiple HPV infections were observed in 22.38% of patients (42).

On the other hand, smoking is an important risk factor for HPV infection. Smoking weakens the immune system, so the body is less able to defend itself against the virus. Also, alcohol consumption increases the risk of getting infected with HPV. We observed that only 6% were alcoholics and 25% were smokers, who had more high-risk genotypes. However, due to the limitations of the studies, there is no significant relationship between smoking and alcohol consumption and HPV infection. Our results were generally inconsistent with previous findings regarding the association between smoking (55), HPV prevalence (56), incidence (57), and persistence (58). currently, it remains unclear how smoking affects HPV infection in women, but there are many possible mechanisms. For instance, *in vivo *research shows that smoking could increase cell proliferation in various tissues and cell types, which in turn could give rise to increased proliferation or production of HPV due to smoking. It has also been shown that the compounds of cigarette smoke change the function of immune cells (59). In addition, the number of births could affect the risk of HPV infection. The more pregnancies and deliveries, the higher the chance of getting HPV. The reason is the possible damage to the vaginal tissues during childbirth, which leads to greater vulnerability (26). Nonetheless, in this study, the HPV prevalence genotypes decreased with the increase in the number of births. On the other hand, a study in 2022 by Hasheminejad *et al.*, indicated that occupation and marital status might be considered risk factors for contracting high-risk types of HPV in Iranian women (60). However, most of the cases with positive HPV were educated.

In sum, the findings indicated that co-infection with several HPV genotypes is common in the studied HPV-positive population, and HPV 6 was reported as the most dominant genotype among patients. This study also reported a significant increase in HPV 52, which is rarely reported in Iran. This data might be functional for developing vaccination. 

This study contains many limitations. Note that we may not have concluded from our study that applies to all Iranian women because we recruited participants in a therapeutic setting. Furthermore, it was not possible to assess the potential correlation between HPV infection and clinical abnormalities using cytological analysis of cervical swabs. Furthermore, some sexual characteristics, such as the age of first sexual activity, sexual partners, homosexual or heterosexual activity, prolonged oral contraception, and hormonal contraceptive use, were not recorded at the time this study was conducted. Additionally, we could not obtain a large enough sample size for the risk factor analysis because we neglected to consider the prevalence of HPV in men. 

Besides, due to the limited sample size, the prevalence of different HPV genotypes obtained in the mentioned study might not reflect the distribution of HPV genotypes in Iran. 

Thus, more extensive studies are needed to confirm the obtained results.

To sum up, the low-risk-HPV genotype distribution in our study is essentially similar to that found in other regions of the world. The two most prevalent forms of high-risk HPV in our investigation were HPV16 and HPV52. In the current study, the current study's findings offer important insights into the frequency of HPV genotypes among Iranian women in Tehran who have genital warts. With this knowledge, efficient plans for diagnosing, managing, and preventing illnesses linked to HPV might be created. Furthermore, this information may contribute to the development of more precise techniques for diagnosing HPV infections, which in turn may lessen the prevalence of HPV-related illnesses in women's health. Currently, the Gardasil 9 vaccine, available to everyone, is effective against most HPV strains. Nonetheless, epidemiological surveillance of HPV infection and associated illnesses is an important area of study for tracking and assessing the global adoption of the three antiviral preventive vaccinations that are now on the market (2-, 4-, and 9-valent vaccines). 

Furthermore, understanding the genotypic distribution of HPV makes it possible to comprehend the true burden and consequences of HPV-related diseases in terms of distribution. In turn, this would provide new therapeutic approaches for the creation of next-generation antiviral vaccines, which would address the drawbacks of the current prophylactic regimen. These drawbacks include low or inadequate follow-up due to high vaccination costs, limited protection against certain high-risk HPVs (only those included in the vaccine), and refusal to vaccinate or late program implementation. Immunization management, high expenses, and a narrow range of antiviral protection are all relevant factors (61). 

Furthermore, another global public health goal should be to provide scientific evidence to retest the number of recurrences and improve the outcomes of the proposed treatments. Lastly, health communication should also play a key role. Standardizing the quality and the quantity of information could lead to increased adherence to the various vaccination awareness campaigns. 

## Conclusion

The results of the present study provide valuable information about the prevalence of HPV genotypes among women with genital warts in Tehran, Iran. This information could be used to develop effective strategies for the prevention and treatment of HPV-related diseases. In addition, this data could help to develop more accurate diagnostic methods for HPV infections, which ultimately help reduce the abundance of HPV-related diseases in women’s health. In conclusion, this study will provide an important insight into the HPV genotypes abundance in women with genital warts in Tehran. 
